# A protocol for a systematic review of the effectiveness of interventions to reduce exposure to lead through consumer products and drinking water

**DOI:** 10.1186/2046-4053-3-36

**Published:** 2014-04-15

**Authors:** Lisa Maria Pfadenhauer, Jacob Burns, Anke Rohwer, Eva Annette Rehfuess

**Affiliations:** 1 Institute for Medical Informatics, Biometry and Epidemiology, University of Munich, Marchioninistrasse 15, 81377 Munich, Germany

## Abstract

**Background:**

The toxic heavy metal lead continues to be a leading environmental risk factor, with the number of attributable deaths having doubled between 1990 and 2010. Although major sources of lead exposure, in particular lead in petrol, have been significantly reduced in recent decades, lead is still used in a wide range of processes and objects, with developing countries disproportionally affected. The objective of this systematic review is to assess the effectiveness of regulatory, environmental and educational interventions for reducing blood lead levels and associated health outcomes in children, pregnant women and the general population.

**Methods/design:**

The databases MEDLINE, Embase and the Global Health Library (GHL) will be searched using a sensitive search strategy. Studies in English, German, French, Spanish, Italian or Afrikaans will be screened according to predefined inclusion and exclusion criteria. We will consider randomized and non-randomized studies accepted by the Cochrane Effective Practice and Organization of Care (EPOC) Group, as well as additional non-randomized studies. Screening of titles and abstracts will be performed by one author. Full texts of potentially relevant studies will be independently assessed for eligibility by two authors. A single author will extract data, with a second reviewer checking the extraction form. Risk of bias will be assessed by two researchers using the Graphical Appraisal Tool for Epidemiological studies, as modified by the Centre for Public Health at the UK National Institute for Health and Care Excellence. Any inconsistencies in the assessment of eligibility, data extraction or quality appraisal will be resolved through discussion. Where two or more studies report the primary outcome blood lead levels within the same population group, intervention category and source of lead exposure, data will be pooled using random effects meta-analysis. In parallel, harvest plots as a graphical method of evidence synthesis will be used to present findings for blood lead levels and secondary outcomes.

**Discussion:**

This systematic review will fill an important evidence gap with respect to the effectiveness of interventions to reduce lead in consumer products and drinking water in the context of new WHO guidelines for the prevention and management of lead poisoning. It will also contribute to setting a future research agenda.

## Background

### Description of the health problem

Lead is a toxic heavy metal that causes extensive environmental contamination due to its environmental persistence and transportability [1]. It has become widely distributed in the environment - especially due to industrialization and mining activities - whereby human exposure has steadily increased [2]. Lead is one of the leading environmental risk factors, with the number of attributable deaths having doubled between 1990 and 2010 [3].

In most developed countries, a clear reduction in blood lead levels (BLL) occurred over the past decades, mainly due to phasing out of leaded petrol. In the US, the prevalence of elevated BLL (≥10 g/dL ) in children aged one to five years decreased from 8.6% in 1988 to 1991 to 1.4% in 1999 to 2004, constituting a 84% decline, with geometric mean BLLs being lowest in non-Hispanic white children (3.1 g/dL to 1.7 g/dL) [4]. Between 1978 and 1988, marked decreases in the average blood lead levels of adults were noted in many countries, including Belgium, the Federal Republic of Germany, New Zealand, Sweden, and the United Kingdom [5]. While cases of acute lead poisoning have become rare in developed countries, continuous exposure to low levels of lead is still a public health issue, especially among ethnic minorities and socio-economically disadvantaged groups [6].

Although awareness is increasing, lead exposure remains a significant public health problem in developing countries, particularly due to the fact that regulations and policies are missing [7]. Differences between major sources of exposure are observed: paint is a major source of lead exposure in the US, while lead-glazed ceramics impose a significant health risk in Latin America [8]. Children living in Africa are still exposed to the highest levels of lead in petrol as well as to lead released by burning of paper products, discarded rubber, battery casings, and painted wood for cooking and heating [9]. In various countries in Africa and South East Asia, as well as in China, lead-based paint is readily available for sale, with 78% of the sample of 80 paints bought in China (100%), India (72%), Malaysia (56%) and Singapore (9%) containing more than 600 ppm of lead [10]. In 2009, an additional sample of 300 lead-based paints was collected, including eight additional countries from Africa, Asia and South America. In all countries, high lead content in paint was observed [11].

### Occurrence of lead

Lead can be found in a variety of natural as well as anthropogenic sources. It has a natural occurrence in the Earth’s crust, and is released by volcanic activities, geochemical weathering and sea spray emissions, as well as by the remobilization of historic sources (soil, sediment, water from mining areas) [12]. Considerable amounts of lead were discharged during industrialization. The blood lead concentration in pre-industrial humans is estimated to have been 0.016 μg/dL, a level which is 50- to 200-fold lower than the lowest reported level in people living in remote regions of the Southern and Northern hemisphere today [13]. In all countries that have banned leaded gasoline, average population blood lead levels have declined rapidly [5]. With respect to anthropogenic sources of lead today, 40% of lead is being used as pure metal, 25% in alloys (for example tin, antimony, bismuth, brass, bronze, steel) and 35% in chemical compounds [14].

Unlike in acute lead intoxication, where the respective source of lead exposure can usually be identified, longer-term, persistent exposure to lead is more complex, due to the large variety of sources and pathways.

#### *Lead in consumer products*

The use of lead in consumer products constitutes a major source of exposure. Lead is added intentionally to certain consumer products for its perceived therapeutic effect, for the coloration it imparts to the products as well as for adding weight to spices sold by weight [15].The US Centers for Disease Control and Prevention (CDC) identified candy, folk and traditional medicines, ceramic dinnerware, children’s jewellery, clothing ornaments, children’s toys, key chains and other metallic or painted objects as well as products including vinyl, plastic and rubber as potential sources of lead [16].

Paint is a common source of exposure to lead [17]. Especially when renovating, dust and chips from chipping or chalking of lead-based paints present a source of lead exposure. Lead paint is contained in window frames, walls, the outside of homes, or other surfaces. Some 80% of the global annual primary (mining) and secondary (recycling) lead consumption occurs in the production of lead batteries or accumulators [18]. Batteries provide a major source of lead poisoning in developing countries, where they are often recycled in small shops or backyards or are illegally disposed of. Toys and cheap jewellery may be painted with lead-based paint or be made of lead-contaminated materials [19,20]. Various cases of contaminated toys, as well as Halloween and Easter products, were reported recently, leading to a large-scale recall of toys imported from China to the US [21]. The CDC, moreover, issued a warning on imported candies. Lead is either added through ingredients such as chili or tamarind, the production process (drying, storing, grinding) or in the wrapping [22]. Chocolate and candy sold in India also tested positive for heavy metals, including lead [23], as was the wrapping in Korea, [24], Taiwan [25] and Mexico [26]. Illicitly distilled beverages (moonshine), which constitute a major source of alcohol intake worldwide (about 30%, [27]) are oftentimes produced in makeshift distilling units containing harmful toxins, such as lead [28]. Lead-glazed earthenware, glasses and other dishes were also reported to be responsible for lead exposure [29], which is of particular relevance in Latin American countries [8]. Moreover, lead is often used in a variety of cosmetics, such as lipsticks and eye cosmetics, including the traditional Indian eye cosmetic ‘surma’ [30,31] or ‘sindoor’ , which is both used as a food additive and as a cosmetic [32]. A European survey of lead content in lipsticks and lip gloss revealed that 49 out of 223 lip articles (22%) sold in 15 EU member states contained more than 1 mg/kg lead [33]. Lead and other heavy metals were detected in traditional Indian Ayurvedic medicines, which are also sold in the US [34-36]. Several cases of lead poisoning caused by unbranded Ayurvedic medicines have been reported [36].

#### *Lead in water*

While water constitutes a relatively minor source of daily lead intake for adults, it may be a greater source of lead exposure for children and bottle-fed infants [37]. According to the US Environmental Protection Agency (USEPA), 20% of the lead exposure of children can be attributed to lead-contaminated water [38], and the concentration of lead in water is correlated with BLL in children [39].

Large concentrations of lead are rarely present in naturally occurring water bodies or treated water [40]. Lead particles, which accumulate in soil, can be flushed out by rain, contaminating rivers, lakes and streams. However, concentrations are relatively low, as is the likelihood of lead contaminating groundwater – except for rainwater being acidic or ‘soft’ (containing few or no minerals) [41].

Yet, elevated lead concentrations in water are often observed as the result of corrosive water effects on materials used in distributing water: plumbing, coating, solder, pipes and pipe joints and fittings [40]. Lead concentrations are determined by various factors, such as pH, temperature, water hardness and standing time of the water, with soft, acidic water being the most plumbosolvent [40,42]. When water distribution systems are irregularly used, the interior of the lead service pipes is exposed to air. Scale that has built up over the years becomes brittle and flakes away. When water flows through the pipes again or when lead service pipes are damaged or undergo maintenance [43], lead is dissolved, causing contamination of drinking water [44].

### Human health response

In contrast to elements such as iron or zinc, which are essential for human nutrition, lead is both non-essential and toxic for the human body [45]. The main routes of exposure are ingestion and inhalation.

Lead is available as organic lead (that is containing carbon) and inorganic lead (that is not containing carbon). While inorganic lead crosses the less-developed blood-brain barrier in children, organic lead penetrates the blood-brain barrier even in adults, leading to encephalopathy, one symptom of severe acute lead poisoning [46]. Inorganic lead entering the human body is not metabolized, but absorbed, distributed and excreted directly. The absorption rate depends on the chemical and physical form, as well as on the condition of the exposed person. Inhaled lead is completely absorbed, while the rate ranges between 10 and 15% for ingested lead. This rate is higher for pregnant women and children where up to 50% is absorbed. Infants and young children absorb about 40 to 50% of ingested water-soluble lead (adults 3 to 10%). Evidence suggests that this amount may increase to 50 to 60% during fasting where lead is ingested on an empty stomach [47].

While the CDC formerly defined levels above 10 μg/dl as elevated concentrations of lead in blood [48], they now use a reference level for children of 5 μg/dL, which is based on the 97.5th percentile of children’s blood lead concentrations in the US [49]. However, no evidence of a threshold exists [50,51].

The human organism can be exposed to high concentrations of lead on an acute basis (that is acute lead poisoning), or to lower concentrations of lead over a period of months or years (that is chronic lead poisoning). Table 1 illustrates the signs and symptoms associated with acute lead poisoning. Chronic lead poisoning, on the other hand, is hard to diagnose, since symptoms are non-specific and similar to those of many other disorders. Evidence on low and moderate exposure to lead points to subtle or subclinical effects, especially affecting the neuropsychological development of children [52].

**Table 1:** **Signs and symptoms associated with lead toxicity [**[14]**]**

**Mild toxicity**	**Moderate toxicity**	**Severe toxicity**
Myalgia and paraesthesia	Arthralgia	Paresis and paralysis
Mild fatigue	General fatigue	Colic
Irritability	Difficulty concentrating	Lead line (blue-black) on gingival tissue
Lethargy	Muscular exhaustibility	Encephalopathy, sometimes leading to seizures, changes in consciousness, coma and death
Occasional abdominal discomfort	Tremor	
	Headache	
	Diffuse abdominal pain	
	Vomiting	
	Weight loss	
	Constipation	

The cumulative toxicant affects neurological, haematological, gastrointestinal, musculoskeletal, cardiovascular, renal and reproductive systems [12,53]. No matter whether lead enters the body through ingestion or inhalation, the subsequent physiological effects are similar.

#### *Neurological response*

The nervous system is particularly sensitive to lead, with damage seeming to be irreversible [54]. Acute, high-level lead exposure (that is blood lead concentrations in excess of 100 μg/dL) presents as acute severe encephalopathy. The effects on the central nervous system (CNS) may include dullness, irritability, poor attention span, headache, muscular tremor, seizures, coma and death in children [55]. Childhood exposure to lead is inversely linked to intellectual abilities, academic achievement, and psychomotor development [56-59]. Exposure to low or moderate lead levels is associated with deficits in attention, language, memory, cognitive flexibility, academic achievement and visual-motor integration, as well as aggression [58-62].

#### *Renal response*

Lead is associated with acute and chronic nephropathy, depending on the exposure period and the level of lead [63]. Chronic nephropathy can lead to chronic renal dysfunction (for example, kidney failure, chronic kidney disease, hyperuricemia and gout) and dysfunction of the immune system [64-67]. Besides, studies have shown an association between blood lead levels and blood pressure (see below), with hypertension being a cardinal feature of lead nephropathy [65]. Recent evidence also points to low-level environmental lead exposure – levels common also in developed countries – being associated with reduced kidney function, even in the absence of other co-morbidities [68].

#### *Reproductive response*

Lead is a reproductive toxicant. In men, lead is associated with impaired spermatogenesis, chromosomal damage, infertility, abnormal prostatic function and changes in serum testosterone. In women, it is associated with miscarriage, premature membrane rupture, pre-eclampsia, pregnancy hypertension and premature delivery. It can also lead to infertility in women [69]. At low-lead exposure, elevations in maternal blood pressure during labour and delivery have been observed [70].

#### *Cardiovascular response*

Even low-level exposure to lead is associated with cardiovascular effects [71,72]. High concentrations of lead, as they occur during occupational exposure, are toxic to both the heart and vascular smooth muscles [73]. Occupational as well as environmental exposures to lead are associated with arterial hypertension [74-77]. Lead poisoning during childhood is likely to cause clinically significant hypertension, which is considered a cause of arterial hypertension [78]. Evidence suggests there is a link with clinical cardiovascular outcomes, such as cardiovascular and coronary heart disease, stroke mortality and peripheral arterial disease [79].

#### *Burden of disease attributable to lead*

The effects of low lead exposures on mortality have been widely studied, especially in large cohort studies [71]. For example, the US National Health and Nutrition Examination Survey (NHANES) showed that adults aged 30 to 74 years with baseline blood lead levels of 20 to 29 μg/dL had 46% increased all-cause mortality (39% increased circulatory mortality, 68% increased cancer mortality) [80]. A 2009 cohort study conducted in the US showed increased mortality, especially from coronary heart disease, in women with BLLs >8 μg/dL [81]. A further 2009 cohort study in an environmentally exposed population with low blood lead levels suggests that bone lead is associated with all-cause and cardiovascular mortality. In this study, bone lead was not associated with cancer, and blood lead was not associated with any mortality category [82].

Globally, around 1% of the total disability-adjusted life years (DALYs) are attributable to lead exposure, with developing countries being disproportionally affected (1.14% compared to 0.72% in developed countries) [83]. Around 1% of global deaths are attributable to lead (651,632 deaths) [84]. Table 2 shows the risk factor attribution of lead to the share of non-communicable diseases; lead appears to be a significant cause of hypertensive heart disease.

**Table 2:** **Global disability-adjusted life years (DALYs) attributable to lead (both sexes, all ages, 2010)**[83]

	**DALYs attributable to disease as % of total DALYs**	**% DALYs attributable to lead**
**Non-communicable diseases**	53.88%	1.04%
Ischemic heart disease	5.23%	4.01%
Haemorrhagic and other non-ischaemic stroke	2.53%	5.90%
Ischaemic stroke	1.59%	4.64%
Other cardiovascular and circulatory diseases	0.69%	2.75%
Hypertensive heart disease	0.62%	9.48%

#### *Vulnerable groups*

Lead exposure is ubiquitous. Thus the whole population is potentially exposed, especially people living in lower socio-economic neighbourhoods, namely in old houses, post-industrial areas or in close proximity to mining and smelting areas or highways.

Children up to six years of age are particularly susceptible to adverse health effects of lead through a combination of greater exposure, greater absorption and retention and greater developmental vulnerability [85]. Increased risk of lead exposure is determined by exposure during pregnancy (lead crosses the placenta) [86,87], increased intake of food, water and air relative to body weight, risk behaviour (for example, hand-to-mouth behaviour), and more time spent in polluted environments (for example, home) [1,37,88,89]. The absorption of lead is four to five times higher in children than in adults (excluding pregnant women), and only 30% of the absorbed lead is excreted, while 70% is accumulated in bone, blood, brain, kidneys, liver and lungs [45]. Finally, exposure may occur during windows of developmental vulnerability, and children have more time to develop late consequences of lead intake.

A similar vulnerability applies to pregnant and nursing women due to the mobilization of lead stored in bones, which applies to women who are chronically exposed to lead or have retained lead from previous lead exposures [90,91]. The bone resorption also presents an endogenous source of lead during lactation where the maternal body responds to the calcium needs of the infant [91]. BLLs change during pregnancy and the postpartum period, with levels being significantly higher postpartum [91]. Lead affects a wide range of processes critical to the development of the CNS, including myelination [92], differentiation [93] and synaptogenesis [94].

### Description of the intervention

Different interventions are available to reduce exposure to lead in consumer products and drinking water. For the purposes of this review, these are categorized as regulatory interventions, environmental interventions and educational interventions.

#### *Regulatory interventions*

Regulatory interventions are legislation and regulations used by governments around the world to protect their citizens against health and safety risks derived from lead-contaminated consumer products and drinking water. Standards for lead have been introduced since the late-1970s, starting off with phasing lead out of petrol. Regulations, product bans and testing requirements intended to prevent acute or chronic exposure to lead in paint, ambient air, drinking water, workplace environments and consumer products followed. These are intended to limit the amount of lead allowed in drinking water, ban the use of lead in solder and plumbing components, and require the replacement of lead pipes in water systems if they fail to meet a specified testing standard. Regulations phasing out or banning uses of lead in consumer products have led to reductions in BLLs [95].

Lead in drinking water has been recognized as an important source of lead intake for decades. The current provisional World Health Organization (WHO) guideline is 10 μg/L [40].

#### *Environmental interventions*

Environmental interventions include but are not restricted to engineering, filtering and treating measures, which aim to eliminate lead exposure by repairing or removing water distribution systems or elements thereof by reducing corrosion and lead-leaching corrosivity in water systems [44]. To our knowledge, there are no environmental interventions aimed at reducing lead in consumer products.

The repairing or removal of leaded pipes is cost-intensive [44], with full lead service line replacement being preferable to partial replacement since the latter might disturb the pipes and knock off lead-bearing pipe scale [96]. Lead can moreover be removed chemically or technically at the water system level, at the point of entry to the residence or at the point of use. Means to remove lead include filters (for example, reverse osmosis filters, distillation), absorbers (for example, zeolites, resins, or activated carbon) [97] and chemicals (for example, phosphoric acid, ortho-phosphoric acid polyphosphate, silicates). Also, chemical additives that increase pH and alkalinity (optimal pH range 7.5 to 9.5; optimal alkalinity range 30 to 75 mg/l) can reduce corrosion and lead solubility and thereby reduce lead concentrations in drinking water [98].

#### *Educational interventions*

Educational interventions can include information on sources of lead in consumer products and drinking water, health sequelae of lead, and lead exposure reduction strategies, including household cleaning, safe use of water, hygiene, and nutrition. These may be directed at protecting both adults and children.

Interventions may include information campaigns that inform parents or the general public about consumer products possibly contaminated with lead, about how to exercise informed product choice and about how to use products correctly.

Interventions aiming at behaviour changes may promote actions, such as letting the water run before use, especially in old buildings, whose plumbing has not been restored in the past 50 years, or avoiding drinking water from the hot tap, since this water may contain higher concentrations of lead than cold water. In order to ensure the safety of baby formula, it should not be prepared with contaminated tap water [99].

### How the intervention might work

**Figure 1:**
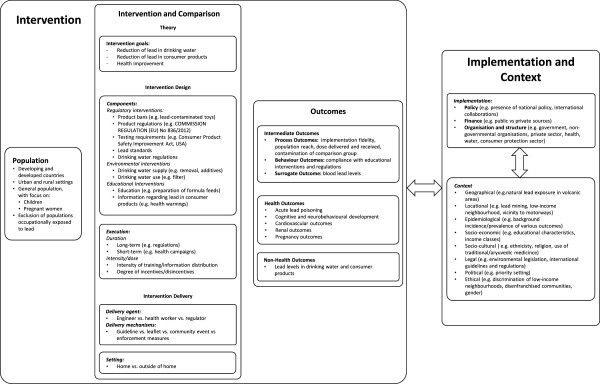
System-based logic model for interventions to reduce exposure to lead through consumer products and drinking water.

Interventions to reduce exposure to lead through consumer products and drinking water must often be carried out over an extended period of time and may involve multiple governmental sectors including environment, transport, water, housing and health. Also, such interventions may not lead to immediate changes in human exposure or health outcomes. This, as well as the complexity of the environmental and biological pathways leading to a health response, complicates the assessment of the effects of such interventions. In order to help understand the relationship between lead interventions and elevated BLLS and to structure and guide the review process, we developed the system-based logic model in Figure 1, based on methodological work undertaken as part of the EU-funded INTEGRATE-HTA project (Anke Rohwer, personal communication).

### Why it is important to do this review

Lead as a ubiquitous pollutant is responsible for a substantial disease burden [66], and disproportionately affects the developing world; 99% of the severest cases of overt lead poisoning occur in developing countries [100]. The health outcomes associated with lead also show stark inequalities in exposure and health effects for disadvantaged groups within industrialized and developing countries. Despite sharp reductions in the general population’s exposure to lead since the 1970s, substantial numbers of ethnic minority and low-income children continue to exhibit unacceptably high BLLs [101,102]. To our knowledge, no systematic review has been undertaken to assess the effectiveness of interventions to reduce lead in consumer products and drinking water, although a systematic review has been conducted for household interventions for preventing domestic lead exposure in children [89]. This review is undertaken in the course of the development of WHO guidelines for the prevention and management of lead poisoning.

### Objectives

The objective of this systematic review is to assess the effectiveness of regulatory, environmental and educational interventions for reducing blood lead levels and associated health outcomes in children, pregnant women and the general population.

## Methods/design

This systematic review is not registered with PROSPERO.

### Criteria for considering studies for this review

#### Types of studies

Due to the range of lead interventions, we will consider both randomized and certain non-randomized studies for this review. In doing so, we will distinguish between those non-randomized studies accepted by the Cochrane Effective Practice and Organization of Care (EPOC) Group [103] and additional non-randomized studies. The following study designs will therefore be eligible for inclusion:

• Individually randomized trials

• Cluster randomized trials

• Controlled before-and-after studies adhering to EPOC standards (CBA-EPOC) – with at least two intervention sites and two control sites

• Interrupted time series studies adhering to EPOC standards (ITS-EPOC) – with at least three data points before and after a clear intervention point

• Controlled before-and-after studies not adhering to EPOC standards (CBA) – with less than two intervention and/or control sites

• Uncontrolled before-and-after studies (UBA)

• Interrupted time series studies not adhering to EPOC standards (ITS) – with less than three data points before and after a clear intervention point

• Repeated cross-sectional studies (CSS) – with a clear intervention point and effect data taken both before and after the intervention

As we expect inconsistencies in naming among studies, we will be very careful not to exclude studies solely based on study design labels. A cohort study, for example, in which a clear intervention point exists and an assessment is undertaken both pre- and post-intervention, is essentially an uncontrolled before-and-after study according to our definitions.

### Types of participants

We will consider studies where children, pregnant women and/or adults are included. We restrict our participants to people who are not occupationally exposed to lead.

### Types of interventions

Interventions aiming to reduce exposure to lead through consumer products (including cans, ceramic ware, jewellery, toys, cosmetics, traditional medicines, paint) and drinking water are categorized according to their respective programmatic approach. Interventions specifically aiming to reduce exposure to leaded paint will be excluded, as their effectiveness was assessed in a recent Cochrane review [89].

• Regulatory interventions (for example, standards for lead, product bans, product testing requirements)

• Environmental interventions (for example, removal of leaded pipes, additives to reduce lead solubility, water filters)

• Educational interventions (for example, restricted use in children, product choice, letting water run before drinking)

### Types of outcomes

#### *Primary outcomes*

The primary outcome of this review is blood lead level. Blood lead levels, measured in μ/dl, can be assessed in venous blood samples or capillary blood samples. Effect estimates may be reported as continuous measures (for example, mean or median blood levels for a given population) or categorically (for example, percentage of population with blood lead levels above a defined threshold).

#### *Secondary outcomes*

Among children, two secondary outcomes will be assessed: the critical short-term outcome will be acute lead poisoning. Symptoms of acute lead poisoning include gastrointestinal (anorexia, nausea, vomiting, abdominal pain, constipation, metallic taste) and nervous system symptoms (poor concentration, headache, fatigue, malaise, language and speech delay, behavioural problems, encephalopathy, ataxia, seizure, coma). Physical examination can reveal signs of raised intracranial pressure, lead lines in teeth, gout and hypertension. The diagnosis of lead poisoning is confirmed through laboratory tests showing elevated blood lead levels, hypochromic anaemia, red blood cells with basophilic stippling, elevated protoporphyrin levels, elevated transaminase levels, and proteinuria, glucosuria and aminoaciduria in urine [15]. An important long-term outcome is cognitive and neurobehavioural development, as measured by standardized measures of intelligence quotient, behaviour and development. These include the Stanford Binet Intelligence Scale, Wechsler Intelligence Scale for Children, Wechsler Preschool and Primary Scale of Intelligence, Kaufman Test of Educational Achievement (K-TEA) for the assessment of intelligence, the Griffiths Mental Development Scales and the Child Behaviour Checklist.

In relation to pregnant women, pregnancy outcomes (miscarriage, stillbirth, premature birth, low birth weight, minor malformations of the embryo [104]) will be assessed.

Among adolescents and adults, outcomes to be documented are hypertension and renal problems, as assessed mainly by the glomerular filtration rate.

### Adverse events

We will also carefully examine included studies for any adverse events reported. An adverse event is defined as any unfavourable outcome that occurs during or after the delivery of the intervention but is not necessarily caused by it [105]. For example, an adverse outcome would be contamination of drinking water with other chemicals as a consequence of water treatment changes aimed at reducing lead concentrations [97].

### Search methods for identification of studies

#### *Sources to search*

Searches will be performed within the following electronic databases:

• MEDLINE

• EMBASE

• Global Health Library

### Designing the search strategy

The databases will be searched using the intervention, intermediary agent and outcome search terms described in Table 3; these groups of search terms will be connected using the Boolean operator AND. Search terms for individual databases will be modified as necessary to meet the requirements of any changes to indexing terms or database platforms. Searches will be conducted in English. The eligibility of studies published in English, German, French, Spanish, Italian or Afrikaans will be assessed by the review team itself; for any studies published in other languages, we will endeavour to identify assistance with their assessment for eligibility. Table 3 describes general search terms, while the explicit search strategy can be found in Additional file 1.

**Table 3:** Search strategy

**Exposure/outcome**	**Intermediary agent**	**Intervention**
‘Blood lead level’	Product	Reduction*
‘blood lead’	Products	Reduce*
‘BLL’	Production	Control
‘B-Pb’	‘consumer product*’	Intervent*
Lead [MeSH]	Can	Regulat*
‘Pb’	Cans	Legislat*
‘Lead poison*’	Jewellery	Politic*
‘Lead poisoning’ [MeSH]	Jewelry	Policy
‘Lead intoxication’	Toy*	Policies
‘Lead toxicity’	Candy	Government*
Plumbism	Candies	‘government regulation’ [MeSH]
Saturnism	Alcohol	Guideline [MeSH]
‘colicapictonum’	Alcoholic	Educat*
‘lead sulphide’	‘ceramic ware’	‘health warning*’
‘lead sulfide’	Glaze*	Inform*
‘lead chloride’	Potter*	
‘lead chromate’	Earthenware*	
‘lead oxide’	Glass*	
‘lead nitrate’	Dish*	
‘lead acetate’	Batter*	
‘Lead expos*’	Accumulator*	
‘Lead hazard*’	Cosmetic*	
‘Lead pollut*’	Lipstick*	
Lead poisoning/prevention & control*	Lip gloss	
	Kohl	
	‘eye shadow’	
	Ayurved*	
	‘traditional medic*’	
	Alternative medicine	
	Alternative remedy	
	Ethnic medicine	
	Ethnic remedy	
	Complementary medicine	
	Herb	
	Herbal	
	Spice	
	Spices	
	Surma	
	Sindoor	
	Water	Filter*
	‘water supply’	‘reverse osmosis’
	‘drinking water’	Absorbing
	Pipe	Absorb
	Pipes	Absorption
		Tap
		Taps
		Remov*
		Engineer*
		Additive*
		Solubility
		Corrosive
		corrosivity
		Acid
		Acidic
		‘pH’
OR	OR	OR

*The asterix denotes truncation: for the truncated term, all possible suffix variations will be retrieved.

### Data collection and analysis

#### *Selection of studies*

Screening of titles and abstracts will be performed by one author, that is one of Lisa Pfadenhauer (LP), Anke Rohwer (AR) and Jake Burns (JB). At this stage, only studies that are clearly not appropriate for inclusion will be excluded. Certain details regarding study design and features are often not as well reported in non-randomized studies when compared with randomized controlled trials. If certain key criteria for inclusion cannot be ascertained from title or abstract, the study will be kept for full-text screening.

Full texts of seemingly relevant studies will be assessed for eligibility by two authors (that is two of LP, AR, JB and Eva Rehfuess (ER)). Disagreements between the two reviewers will be resolved through discussion, and a third reviewer will be consulted where necessary. Reviewers will document reasons for exclusion.

#### *Data extraction and management*

LP, JB or AR will extract data using a tabular extraction form. The data extraction form will be checked by a second reviewer. Inconsistencies or disagreements between reviewers will be resolved through discussion, and ER will be consulted where necessary. We will record information and effect estimates for all primary and secondary outcomes reported by the study.As significant differences between interventions are expected, we will focus on extracting all relevant data to thoroughly describe the intervention. For each intervention, we will extract the intervention components (related to technology and infrastructure, education, regulations and policies as specified in the logic model in Figure 1) as well as intervention execution (dose, duration, intensity and timing) and delivery aspects (level of implementation, delivery agent, organization and structure).

Relevant contextual and implementation data, based on a context and implementation framework developed as part of the EU-funded INTEGRATE-HTA project (Lisa Pfadenhauer, personal communication), will be extracted, where available. This framework places important contextual domains within the setting, community, national and international level. These eight domains are:

• locational

• geographical

• epidemiological

• socio-economic

• socio-cultural

• political

• legal

• ethical

Also, data that could highlight possible health inequality issues arising during implementation or any time thereafter will be extracted. This will be based upon the PROGRESS framework [106], which includes place, race, occupation, gender, religion, education and socio-economics.

#### *Quality appraisal of included studies*

The quality of included studies will be independently assessed by two researchers (two of LP, JB, AR), using the modified Graphical Appraisal Tool for Epidemiological studies (GATE) tool [107], as tailored to public health questions and applied by the Centre for Public Health at the UK National Institute for Health and Care Excellence (NICE) [108]. The two versions of this tool – one for quantitative intervention studies, one for quantitative studies reporting correlations and associations – can be applied across all experimental and observational study designs.

For intervention studies, the appraisal checklist comprises key aspects exerting influence on internal and external validity:

• characteristics of study participants

• definition of, and allocation to, intervention and control conditions

• outcomes assessed over different time periods

• methods of analyses.

The checklist embraces five sections. Section 1 aims to describe key population criteria in order to assess the external validity of the study, while Sections 2 to 5 determine the internal validity of the study. The wording of the checklist items allows five responses, as shown in Table 4.

**Table 4:** Possible responses to individual items in modified Graphical Appraisal Tool for Epidemiological studies (GATE)

	
**++**	Indicates that for that particular aspect of study design, the study has been designed or conducted in such a way as to minimise the risk of bias.
**+**	Indicates that either the answer to the checklist question is not clear from the way the study is reported, or that the study may not have addressed all potential sources of bias for that particular aspect of study design.
**-**	Should be reserved for those aspects of the study design in which significant sources of bias may persist.
**Not reported (NR)**	Should be reserved for those aspects in which the study under review fails to report how they have (or might have) been considered.
**Not applicable (NA)**	Should be reserved for those study design aspects that are not applicable given the study design under review (for example, allocation concealment would not be applicable for case control studies).

GATE, Graphical Appraisal Tool for Epidemiological Studies.

Interrupted time series, uncontrolled before-and-after studies and repeated cross-sectional studies will be treated as quantitative studies reporting correlations and associations. The modified GATE for these studies resembles the modified GATE for intervention studies, but emphasizes selection of the exposure group and statistical control for confounding rather than intervention allocation and blinding [108].

Each intervention and correlational study is then awarded an overall rating of likely internal and external validity, based on the criteria depicted in Table 5.

**Table 5:** Overall rating of internal and external validity in modified Graphical Appraisal Tool for Epidemiological studies (GATE)

	
**++**	All or most of the checklist criteria have been fulfilled, where they have not been fulfilled the conclusions are very unlikely to alter.
**+**	Some of the checklist criteria have been fulfilled, where they have not been fulfilled, or not adequately described, the conclusions are unlikely to alter.
**-**	Few or no checklist criteria have been fulfilled and the conclusions are likely or very likely to alter.

GATE, Graphical Appraisal Tool for Epidemiological Studies.

#### *Measures of treatment effect*

We will present dichotomous data using risk ratios. For continuous data using arithmetic means, geometric means or medians, we will present mean or median differences. All results will be presented with their associated 95% confidence intervals.

#### *Unit of analysis issues*

If the included cluster randomized controlled trials have sufficiently accounted for the cluster design, we will include the effect estimates in the meta-analysis, combining them with individually randomized trials. If clustering has not been addressed, we will attempt to adjust the data for clustering by inflating the standard errors by multiplying them by the square root of the design effect [105]. We will then include the adjusted effect estimates in the meta-analysis.

#### *Dealing with missing data*

In the case that missing information on study features, intervention characteristics or outcome data prevent further use of a study, investigators will be contacted.

#### *Assessment of heterogeneity*

Issues of clinical and methodological heterogeneity will be assessed in tabular form for studies in each of the three intervention categories, with the documentation of the following study-specific characteristics:

• Methods: study design, group assignment, exposure assessment, outcome assessment, result of critical appraisal

• Participants: setting (industrialized vs. developing; urban vs. rural), age

• Context and implementation: based on the above mentioned context and implementation for complex interventions framework (Lisa Pfadenhauer, personal communication).

Statistical heterogeneity will be assessed with an I^2^ calculation in Revman 5.2 [109]. An I^2^ value greater than 50% will be considered substantial, and will be considered statistically significant if the *P* value for the chi^2^ test is <0.1.

#### *Assessment of reporting biases*

We will assess likely reporting biases through the use of funnel plots for outcomes that are reported in more than 10 studies.

#### *Data synthesis*

Where at least two studies report the same primary outcome within the same population group (that is children, pregnant women, adults), intervention category and source of lead exposure, we will pool data using meta-analysis. For studies with multiple comparison groups, only those comparisons assessing an intervention/intervention component compared with no intervention/intervention component will be analysed. In the main analysis, we will pool across all study designs.

Due to expected differences in intervention components and complexity, setting and study population, random-effects models will be implemented for all meta-analyses independent of substantial or limited statistical heterogeneity. Inverse-variance random-effects meta-analyses will be carried out using Revman 5.2 [109]. Effects will be considered statistically significant where a *P* value of less than 0.05 is found.

As we expect much of the evidence to be too heterogeneous for statistical pooling, harvest plots will be developed in parallel to meta-analysis. Harvest plots are a novel form of synthesizing evidence – especially in systematic reviews of complex interventions [110,111] and have been shown to be effective, clear and transparent [112]. Harvest plots will be used to graphically synthesize evidence based on all study designs for the effects of regulatory, environmental and educational intervention categories across all primary and secondary outcomes, where the direction of effect is illustrated by columns (left-hand column: favours control, middle column: no differences, right-hand column: favours intervention).

Two sets of harvest plots will be created. The first set will portray the primary outcome BLL with the intervention category and source of lead exposure shown in rows (that is environmental interventions to reduce lead in consumer products, environmental interventions to reduce lead in drinking water, and so on). Each study is represented by a bar, and identified by the first three letters of the author’s last name. The colours indicate the study population (black for children, grey for pregnant women, and white for the general population). Also illustrated, by height of bar, will be appropriateness of study design with randomized and cluster randomized trial being assigned the greatest height, followed by EPOC-recognized designs (intermediate height) and other non-randomized designs (lowest height). The symbols employed with the modified GATE (++, +, -) will indicate the degree of internal validity for each study.

The second set will develop separate harvest plots for each intervention category in relation to exposure, presenting findings for all primary and secondary outcomes. Here separate rows will present the primary outcome BLL and the five secondary outcomes (that is acute lead poisoning, cognitive and neurobehavioural outcomes, pregnancy outcomes, hypertension and renal outcomes). Harvest plots will be created in Microsoft PowerPoint.

#### *Subgroup analysis*

In order to assess possible sources of heterogeneity, subgroup analyses for the primary outcome BLL may be performed for drinking water interventions (intervention through water authority or at household level) and in relation to context characteristics, although we expect that lack of data will prevent these from being conducted.

#### *Sensitivity analysis*

Where possible, we will conduct the following sensitivity analyses for the primary outcome BLL in relation to study design:

• Meta-analysis based on lower risk of bias study designs only, that is individually and cluster randomized trials as well as CBA-EPOC and ITS-EPOC studies.

• Harvest plots based on lower risk of bias study designs only, that is individually and cluster randomized trials as well as CBA-EPOC and ITS-EPOC studies.

Data permitting, we will also dichotomize continuous measures of BLL using 5 μg/dl (or the threshold most widely used across included studies) as the threshold value, and pool all data for this new dichotomous outcome.

The insights derived from these sensitivity analyses will be compared to those gained through the main synthesis.

## Discussion

This systematic review will fill an important evidence gap with respect to the effectiveness of interventions to reduce lead in consumer products and drinking water in the context of new WHO guidelines for the management and prevention of lead poisoning. It will also contribute to setting a future research agenda.

## Abbreviations

BLL: blood lead level; CBA: controlled before-and-after study; CDC: Center for Disease Control and Prevention; CNS: central nervous system; CSS: cross-sectional studies; DALY: disability-adjusted life years; GATE: Graphical Appraisal Tool for Epidemiological Studies; GFR: glomerular filtration rate; EPOC: Cochrane Effective Practice and Organization of Care; ITS: interrupted time series study; K-TEA: Kaufman Test of Educational Achievement; NHANES: National Health and Nutrition Examination Survey; NICE: National Institute for Health and Care Excellence; UBA: uncontrolled before-and-after study; USEPA: US Environmental Protection Agency; WHO: World Health Organization.

## Competing interests

The authors declare that they have no competing interests.

## Authors’ contributions

LP drafted this protocol in constant exchange with ER. JB, AR and LP jointly developed the method section under revision of ER. AR and LP jointly developed the logic model. All authors read and approved the final protocol.

## Supplementary Material

Additional file 1 **Search Strategies.** Description of data: the additional file provides the explicit search strategy employed for the systematic search in EMBASE, MEDLINE and the Global Health Library. Click here for file
