# Supplementary material for: A protocol for a systematic review of the effectiveness of interventions to reduce exposure to lead through consumer products and drinking water
Source: Syst Rev. 2014 Apr 15;3:36. doi: 10.1186/2046-4053-3-36 (PMC4049510; doi:10.1186/2046-4053-3-36)
Supplement: Additional file 1 — Search Strategies. Description of data: the additional file provides the explicit search strategy employed for the systematic search in EMBASE, MEDLINE and the Global Health Library. [file 2046-4053-3-36-S1.docx]

# SEARCH STRATEGIES

## **Global Health Library**

("Lead" OR "Lead poisoning" OR "Lead poisoning/blood" OR "Lead/adverse effects" OR "Lead/blood" OR "Lead Poisoning/epidemiology" OR "Lead/analysis" OR "Lead poisoning/etiology" OR "Lead Poisoning/prevention & control" OR "Lead toxicity") AND (("water" OR "drinking water" OR "Water supply" OR "Water monitoring") OR ("Household Articles" OR "Household Products" OR "Industry" OR "Manfactured Materials" OR "Cosmetics" OR "Cosmetics/adverse effects" OR "Condiments/adverse effects" OR "Candy/adverse effects") OR ("Medicine, Traditional")) AND (("Consumer Product Safety" OR "Consumer Product Safety/standards" OR "Quality Control" OR "public Policy" OR "Maximum Allowable Concentration" OR " Practice Guidelines as Topic" OR "Health Education") OR ("Sanitary Engineering" OR "Quality Improvement" OR "Chemical Engineering" OR "Water Treatment" OR "Water Monitoring" OR "Engineering" OR "Water supply/standards" OR "Water/Chemistry"))

## **MEDLINE**

| 1 | exp Lead/ |
| --- | --- |
| 2 | Pb.ti,ab. |
| 3 | (Lead adj (sulphide or sulfide or chloride or chromate or oxide or nitrate or acetate)).ti,ab. |
| 4 | exp Lead poisoning/ or (blood lead or BLL or B-Pb or lead poison$ or lead intoxication or lead toxicity or plumbism or saturnism or colica pictonum).ti,ab. |
| 5 | (lead adj (exposed or exposure or hazard$ or polluted or pollution)).ti,ab. |
| 6 | or/1-5 |
| 7 | exp Household Products/ or exp Household Articles/ or exp Cosmetics/ or (Product or Products or production or consumer product* or can or cans or jewellery or jewelry or toy$ or candy or candies or alcohol or alcoholic or ceramic ware$ or glaze$ or potter$ or earthenware$ or glass$ or dish$ or battery or batteries or accumulator$ or lipstick$ or lipgloss or kohl or eye shadow$).ti,ab. |
| 8 | exp Medicine, Traditional/ or (ayurved$ or traditional medic$ or alternative medic$ or alternative remed$ or ethnic medic$ or ethnic remed$ or complementary medic$ or herb or herbal or spice or spices or condiment$ surma or sindoor).ti,ab. |
| 9 | exp water/ |
| 10 | exp drinking water/ |
| 11 | (water adj (system or authorit$ or suppl$ or pipe$ or service line$)).ti,ab. |
| 12 | or/7-11 |
| 13 | exp government regulation/ or exp Consumer Product Safety/ or (guideline or reduction$ or reduc$ or control or intervention$ or regulation$ or regulator$ or legislat$ or politic$ or policy or policies or government$ or education or educational or health warning$ or information or test$ or ban$ or phase-out or phase out or phasing out or repair$ or replac$ or upgrad$).ti,ab. |
| 14 | (lead adj8 (solubility or remov$ or absorb$)).ti,ab. |
| 15 | (water adj8 (filter$ or additive$ or acid$ or treating or treatment$ or monitor$)).ti,ab. |
| 16 | ((pipe$ or solder or plumbing or fixture$ or faucet$) adj8 (remov$ or corrosive$ or corrosivity or corrosion or engineer$)).ti,ab. |
| 17 | 13 or 14 or 15 or 16 |
| 18 | 6 and 12 and 17 |
| 19 | (randomized controlled trial or controlled clinical trial or comparative study).pt. |
| 20 | exp intervention studies/ or exp evaluation studies/ or exp program evaluation/ |
| 21 | exp random allocation/ or exp clinical trial/ or exp single-blind method/ or exp double-blind method/ or exp control groups/ |
| 22 | (randomized or randomised or placebo or randomly or groups).ti,ab. |
| 23 | trial.ti,ab. |
| 24 | (time adj series).ab,ti. or (interrupted* adj2 series).ti,ab. |
| 25 | quasi-experiment$.ti,ab. |
| 26 | (pre test or pretest or pre-intervention or post-intervention or posttest or post test).ti,ab. |
| 27 | (controlled before or "before and after stud$" or follow-up- assessment).ti,ab. |
| 28 | ((evaluat$ or intervention or interventional or treatment) and (control or controlled or study or program$ or comparison or "before and after" or comparative)).ti,ab. |
| 29 | ((intervention or interventional or process or program) adj8 (evaluat$ or effect$ or outcome$)).ti,ab. |
| 30 | (program or programme or secondary analys$).ti,ab. |
| 31 | (Case study or observational study or cohort or uncontrolled study or observational research).ti,ab. or exp Epidemiologic Studies/ |
| 32 | ecological study.ti,ab. |
| 33 | or/19-32 |
| 34 | exp animals/ not humans.sh. |
| 35 | 33 not 34 |
| 36 | 18 and 35 |

## **Embase**

| 1 | exp Lead/ |
| --- | --- |
| 2 | Pb.ti,ab. |
| 3 | (Lead adj (sulphide or sulfide or chloride or chromate or oxide or nitrate or acetate)).ti,ab. |
| 4 | exp Lead poisoning/ or (blood lead or BLL or B-Pb or lead poison$ or lead intoxication or lead toxicity or plumbism or saturnism or colica pictonum).ti,ab. |
| 5 | (lead adj (exposed or exposure or hazard$ or polluted or pollution)).ti,ab. |
| 6 | or/1-5 |
| 7 | exp Household Products/ or exp Household Articles/ or exp Cosmetics/ or (Product or Products or production or consumer product* or can or cans or jewellery or jewelry or toy$ or candy or candies or alcohol or alcoholic or ceramic ware$ or glaze$ or potter$ or earthenware$ or glass$ or dish$ or battery or batteries or accumulator$ or lipstick$ or lipgloss or kohl or eye shadow$).ti,ab. |
| 8 | exp Medicine, Traditional/ or (ayurved$ or traditional medic$ or alternative medic$ or alternative remed$ or ethnic medic$ or ethnic remed$ or complementary medic$ or herb or herbal or spice or spices or condiment$ surma or sindoor).ti,ab. |
| 9 | exp water/ |
| 10 | exp drinking water/ |
| 11 | (water adj (system or authorit$ or suppl$ or pipe$ or service line$)).ti,ab. |
| 12 | or/7-11 |
| 13 | exp government regulation/ or exp Consumer Product Safety/ or exp water monitoring/ or (guideline or reduction$ or reduc$ or control or intervention$ or regulation$ or regulator$ or legislat$ or politic$ or policy or policies or government$ or education or educational or health warning$ or information or test$ or ban$ or phase-out or phase out or phasing out or repair$ or replac$ or upgrad$).ti,ab. |
| 14 | (lead adj8 (solubility or remov$ or absorb$)).ti,ab. |
| 15 | (water adj8 (filter$ or additive$ or acid$ or treating or treatment$ or monitor$)).ti,ab. |
| 16 | ((pipe$ or solder or plumbing or fixture$ or faucet$) adj8 (remov$ or corrosive$ or corrosivity or corrosion or engineer$)).ti,ab. |
| 17 | or/14-16 |
| 18 | 6 and 12 and 17 |
| 19 | (randomized controlled trial or controlled clinical trial or comparative study).pt. |
| 20 | exp intervention studies/ or exp evaluation studies/ or exp program evaluation/ |
| 21 | exp random allocation/ or exp clinical trial/ or exp single-blind method/ or exp double-blind method/ or exp control groups/ |
| 22 | (randomized or randomised or placebo or randomly or groups).ti,ab. |
| 23 | trial.ti,ab. |
| 24 | (time adj series).ab,ti. or (interrupted* adj2 series).ti,ab. |
| 25 | quasi-experiment$.ti,ab. |
| 26 | (pre test or pretest or pre-intervention or post-intervention or posttest or post test).ti,ab. |
| 27 | (controlled before or "before and after stud$" or follow-up- assessment).ti,ab. |
| 28 | ((evaluat$ or intervention or interventional or treatment) and (control or controlled or study or program$ or comparison or "before and after" or comparative)).ti,ab. |
| 29 | ((intervention or interventional or process or program) adj8 (evaluat$ or effect$ or outcome$)).ti,ab. |
| 30 | (program or programme or secondary analys$).ti,ab. |
| 31 | (Case study or observational study or cohort or uncontrolled study or observational research).ti,ab. or exp Epidemiologic Studies/ |
| 32 | ecological study.ti,ab. |
| 33 | or/19-32 |
| 34 | exp animals/ not humans.sh. |
| 35 | 33 not 34 |
| 36 | 19 and 35 |
